# Environmental factors driving fungal distribution in freshwater lake sediments across the Headwater Region of the Yellow River, China

**DOI:** 10.1038/s41598-018-21995-6

**Published:** 2018-02-28

**Authors:** Jianqing Tian, Dan Zhu, Jinzhi Wang, Bing Wu, Muzammil Hussain, Xingzhong Liu

**Affiliations:** 1 State Key Laboratory of Mycology, Institute of Microbiology, Chinese Academy of Sciences, Beijing, 100101 China; 2 CAS Key Laboratory of Mountain Ecological Restoration and Bioresource Utilization & Ecological Restoration and Biodiversity Conservation Key Laboratory of Sichuan Province, Chengdu Institute of Biology, Chinese Academy of Sciences, Chengdu, 610041 China; 3 Zoige Peatland and Global Change Research Station, Chinese Academy of Sciences, Hongyuan, 624400 China; 4 Beijing Key Laboratory of Wetland Services and Restoration, Institute of Wetland Research, Chinese Academy of Forestry, Beijing, 100091 China

## Abstract

Dispersal limitation and environmental filtering are two primary processes involved in shaping microbial community structure. The pristine environmental and geographical relatively isolation of small lakes distributed in the Headwater Region of Yellow River (HRYR) offer a unique opportunity to test the relative roles of these two processes on fungal communities. Here, we investigated the fungal community in sediment samples from 10 lakes located in the HRYR using high-throughput sequencing. The results showed that the fungal community was dominated by Sordariomycetes, Leotiomycetes, Dothideomycetes, Pezizomycetes and Agaricomycetes. The results revealed that altitude, mean annual temperature, C/N ration, dissolve organic carbon and total nitrogen were the best predictors for shaping fungal community structure in these lakes. Significant spatial and environmental distance decay relationships in the fungal community were detected. The partial Mantel test indicated that the fungal community structure was significantly correlated with environmental distance but not with geographic distance. Overall, environmental filtering plays a more important role than dispersal limitation in fungal community structure at a local scale in such an pristine and isolated region.

## Introduction

Growing evidence indicates that fungal diversity and communities can alter a number of crucial ecosystem processes such as decomposition, nutrient cycling and productivity^1^. Understanding fungal biogeography and their underlying processes are essential for predicting the quality of ecosystem services under climate change^2,3^. Many studies have revealed that fungi display biogeographic patterns across spatial scales from centimeters to continents^4–8^. It has been reported that patterns of fungal diversity are controlled by dispersal limitation and environmental filtering^5,9,10^. However, the relative importance of these two factors and how they interact on different spatial scales with fungal communities remains unclear.

Several recent studies demonstrated that the processes controlling fungal communities vary by fungal groups, ecosystem types, and landscape heterogeneity or integrity. While dispersal limitation is a crucial in determining ectomycorrhizal fungal diversity at 10 m^2^ to 10,000 m^2^ scale^11^, environmental heterogeneity drives mycorrhizal fungal community variation at a finer scale^5^. On the other hand, the biogeography of arbuscular mycorrhizal fungi is driven by a combination of dispersal limitation and environment filtering^12–14^. Furthermore, the spatial influence on a fungal community is more pronounced in a contiguous site than in a disjointed site^4^. Therefore, to study integral fungal communities in varied ecosystems is essential to parse out the relative contributions of dispersal limitation and environmental filtering factors to fungal diversity.

Headwater streams mainly arise from a variety of sources including lakes, wetlands, seeps and springs^15^, which are critical sites for nutrient cycling, maintaining the function, and health of whole river networks^16,17^. In several studies, it was postulated that headwaters are critical reservoirs of microbial diversity^18–20^, which may act as repositories and distribution centers of microbes from terrestrial sources^9,18–20^. Studies of invertebrate communities in headwaters exhibit higher β diversity compared with mid-sized steams^21,22^. These patterns may be attributed to large environmental variation among headwaters^23^, but their spatial isolation limit dispersal^22^. Recent works on the headwater communities suggest that the interaction between environmental selection and mass effects were the main driver of biogeographic patterns of a bacterial community^9,18,24,25^. However, whether fungi display a biogeographic distribution signature in headwater lakes, and what are the main factors that shape the fungal community was not addressed in previous studies and is the key subject here.

**Figure 1:**
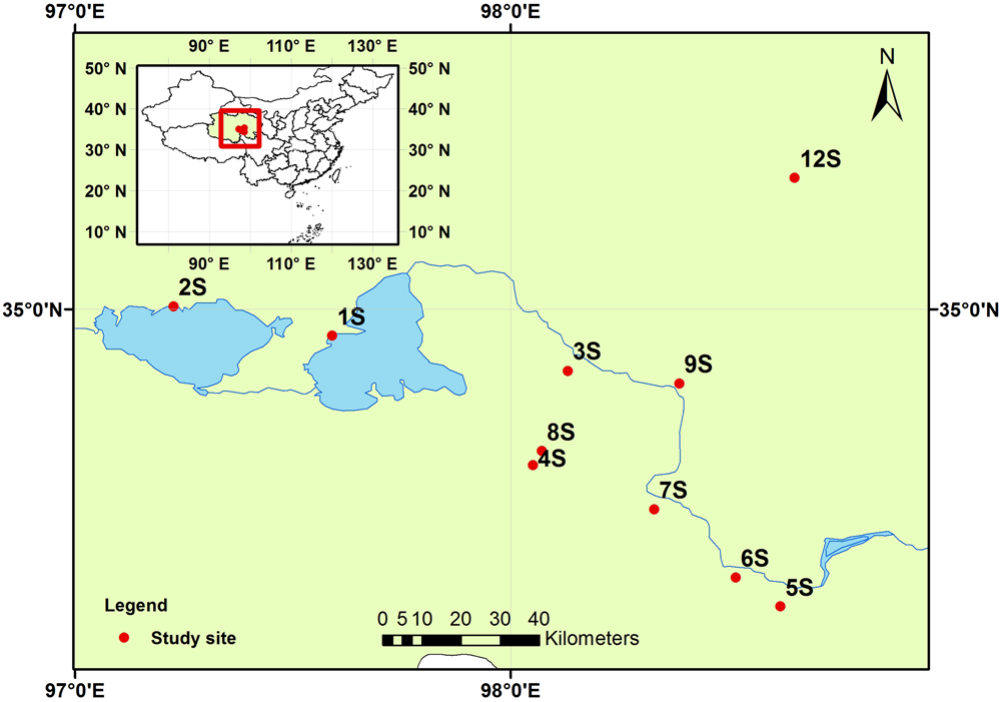
Ten sampling sites across the Headwater Region of the Yellow River, Qinghai-Tibetan Plateau, China. Detailed information of sampling sites is listed in Table 1. The figure was generated using Arcgis 10.3 software (http://www.esri.com/arcgis/about-arcgis).

The Qinghai-Tibet Plateau (QTP), “the Earth’s third pole” and “the water tower of Asia,” is the headwater of ten of the continent’s largest rivers^3,26^. The Headwater Region of the Yellow River (HRYR) is located in northeastern QTP and has approximately 5,300 lakes situated at an average elevation of 4,200 m above sea level^27^. Surrounded by terrestrial matrices, headwater lakes are, to some extent, isolated aquatic habitats^28^ and often connected by the overland dispersal of their biota^29^, where lacustrine bacterial communities are expected to show spatial patterns forged by dispersal restriction^28–31^. However, it is unclear whether similarities among fungal communities decline with increasing fungi exhibit increased community dissimilarity with geographic distances. Compared to plain lakes, plateau headwater lakes are relatively isolated and smaller in area, and more sensitive to anthropogenic activities due to their poor water exchange^32^. Previously, QTP lakes were mainly focused for studying bacterial communities in alkaline lakes^32,33^. However, information on fungal community in sediment of plateau headwater is still very limited. Here, we sampled sediments from 10 freshwater lakes in the HRYR (Fig. 1) and used 454 Life Sciences pyrosequencing to investigate fungal diversity and community composition. The aims of this study were (i) to explore the fungal diversity and composition of the freshwater lake sediments in HRYR, (ii) to determine key factors that shape the fungal distribution among communities and (iii) to quantify the relative importance of environmental filtering and dispersal limitation on fungal community variation.

## Results

### Sediment geochemical characteristics

The major geographical and physicochemical characteristics of the sediments are listed in Table 1. Sediment pH varied from 7.71 to 8.84. Pairwise distances between sampled lakes were ranged from 4 to 148 km. There were significant unimodal trends of TN and dissolved organic carbon (DOC) with varying altitude (R^2^ = 0.24 and R^2^ = 0.28, respectively, *P* < 0.05). The C:N ratio were ranged from 16.3 to 23.0 across all study lakes.

**Table 1:** Characteristics of sampling sites and soil properties in sediment samples from the headwater region of the Yellow River

Site code	Latitude	Longitude	Altitude (m)	pH	TC (g/kg)	DOC (g/kg)	TN(g/kg)	MAT (°C)	Lake area (km^2^)	Shannon index	Chao1	OTU number
1 S	34°56′29.64″	97°35′17.23″	4284	8.34	25.2 ± 3.2	22.6 ± 2.2	1.3 ± 0.2	−4.6	610.7	2.7 ± 0.5	123.9 ± 11.4	111 ± 9
2 S	35°00′26.28″	97°13′32.17″	4299	8.33	17.9 ± 9.5	15.4 ± 5.9	0.9 ± 0.1	−4.6	526	2.6 ± 0.2	140.8 ± 74.5	133 ± 49
3 S	34°51′22.30″	98°07′52.26″	4219	8.04	30.9 ± 7.5	26.3 ± 4.6	1.7 ± 0.3	−4.1	4.7	2.7 ± 0.1	87.2 ± 12.7	77 ± 5
4 S	34°38′38.45″	98°03′04.66″	4221	7.97	41.2 ± 9.4	34.6 ± 2.1	2.2 ± 0.2	−4.1	2.9	3.0 ± 0.2	94.0 ± 6.1	80 ± 2
5 S	34°19′09.43″	98°37′13.24″	4179	7.93	34.8 ± 2.6	31.2 ± 1.5	1.9 ± 0.1	−4.0	33.1	3.1 ± 0.1	139.5 ± 21.3	112 ± 11
6 S	34°23′09.66″	98°31′02.06	4186	7.71	13.7 ± 7.5	11.8 ± 4.5	0.7 ± 0.3	−4.0	15	2.4 ± 0.3	70.4 ± 10.3	59 ± 5
7 S	34°32′32.80″	98°19′52.50″	4200	7.93	17.2 ± 3.0	14.7 ± 1.9	1.0 ± 0.0	−4.1	22.7	2.3 ± 0.1	105.5 ± 23.9	72 ± 2
8 S	34°40′33.46″	98°04′17.92″	4225	8.01	34.0 ± 15.8	29.2 ± 9.5	1.7 ± 0.5	−4.1	19	2.6 ± 0.4	116.8 ± 39.3	83 ± 11
9 S	34°49′53.56″	98°23′16.47″	4218	8.13	39.7 ± 20.3	33.8 ± 12.5	1.9 ± 0.7	−4.1	10.5	2.9 ± 0.1	92.4 ± 24.6	76 ± 8
12 S	35°18′09.01″	98°39′09.65″	4090	8.84	7.28 ± 4.8	6.3 ± 2.9	0.4 ± 0.2	−4.1	6	2.8 ± 0.3	118.2 ± 30.9	102 ± 19

TC: Total carbon; DOC: dissolve organic carbon; TN: total nitrogen; MAT: mean annual temperature.

### Distribution of fungal taxa and phylotypes

**Figure 2:**
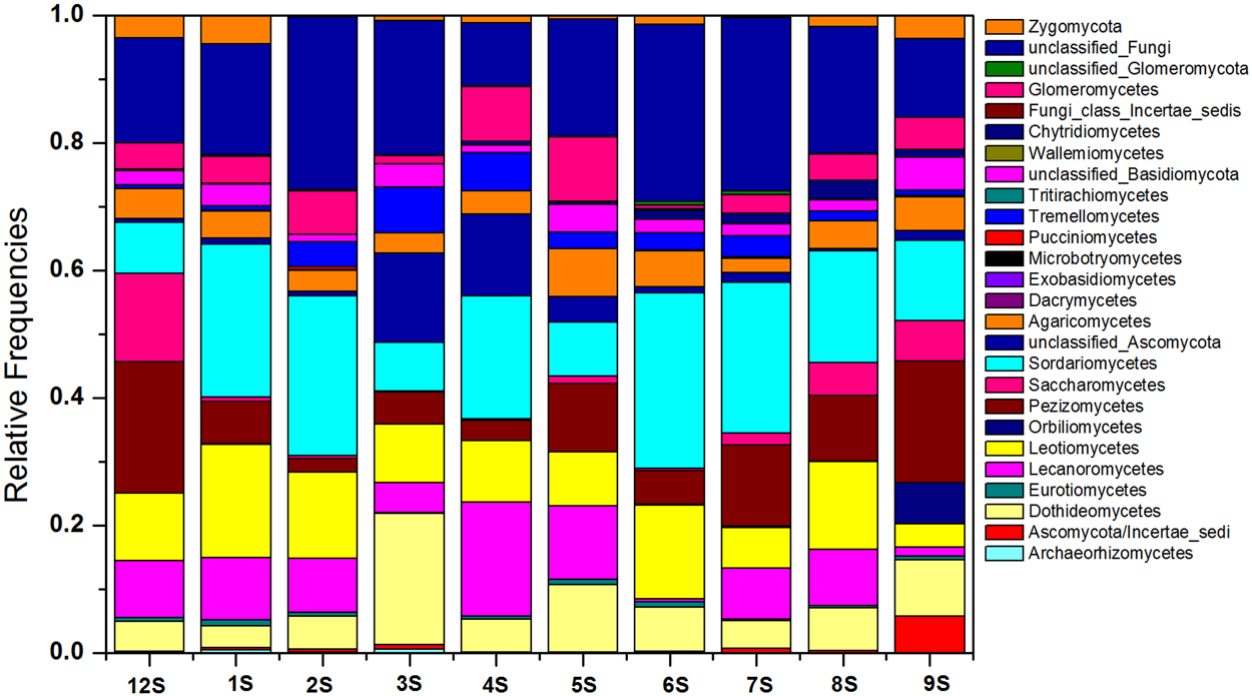
Percent abundance of fungal taxa detected in soil sediments from lakes distributed in the headwater region of the yellow river.

A total of 163,471 quality sequence reads were obtained from 33 samples and assigned to 479 operational taxonomic units (OTUs). These were representative of twenty-four fungal classes belonging to five fungal phyla (Fig. 2). The class that presented the highest diversity was Agaricomycetes (81 OTUs), followed by Dothideomycetes (68 OTUs) and Sordariomycetes (61 OTUs). The abundance of the OTUs in the dataset varied from 0.0006% to 13.1%. Among the total 479 OTUs, twenty-one were shared between all sites and accounted for a relative abundance between 46% and 86%. A class Sordariomycetes predominated across all sampled lakes with abundance ranging from 8.1–37.6%, followed by Leotiomycetes (4.1–36.0%), Dothideomycetes (4.2–25.6%), Pezizomycetes (1.4–25.0%) and Agaricomycetes (3.0–6.0%) (Fig. 2).

### Dynamics of fungal diversity and community

**Figure 3:**
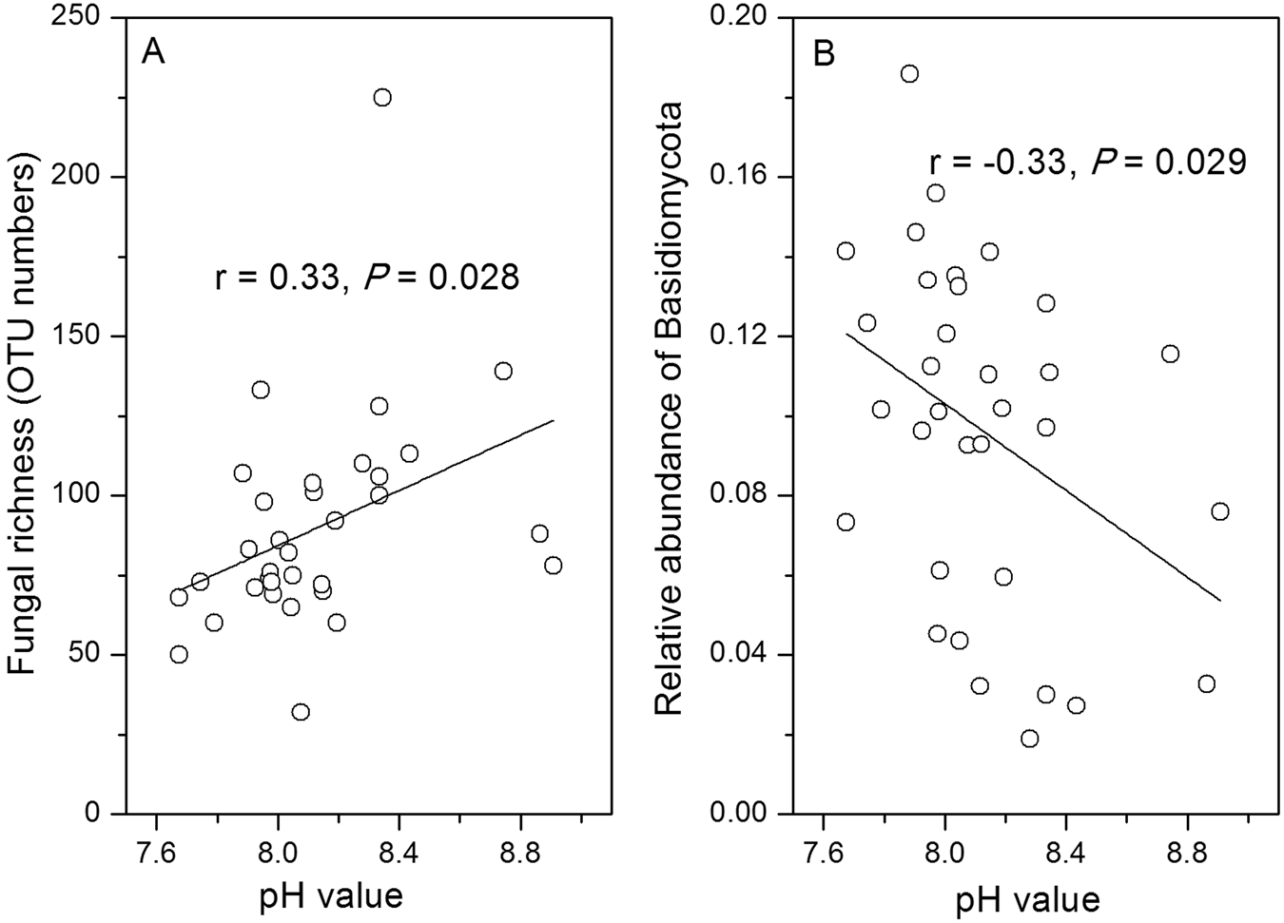
Relationships between fungal richness, relative abundances of fungal groups and sediment pH. (**A**). Relationship between sediment pH and fungal richness (i.e OTU numbers). (**B**) Relationship between sediment pH and relative abundance of Basidiomycota. Linear regressions were used to test Pearson correlation between each taxon’s relative abundance and pH.

The fungal Shannon index was not related to local environmental variables, while fungal diversity slightly increased with warming temperature (linear regression, R^2^ = 0.09, *P* = 0.051). pH was positively correlated with fungal OTUs richness (linear regression, *r* = 0.33, *P* = 0.028) (Fig. 3A), but negatively correlated with Basidiomycota abundance (linear regression, *r* = − 0.33, *P* = 0.028) (Fig. 3B).

**Figure 4:**
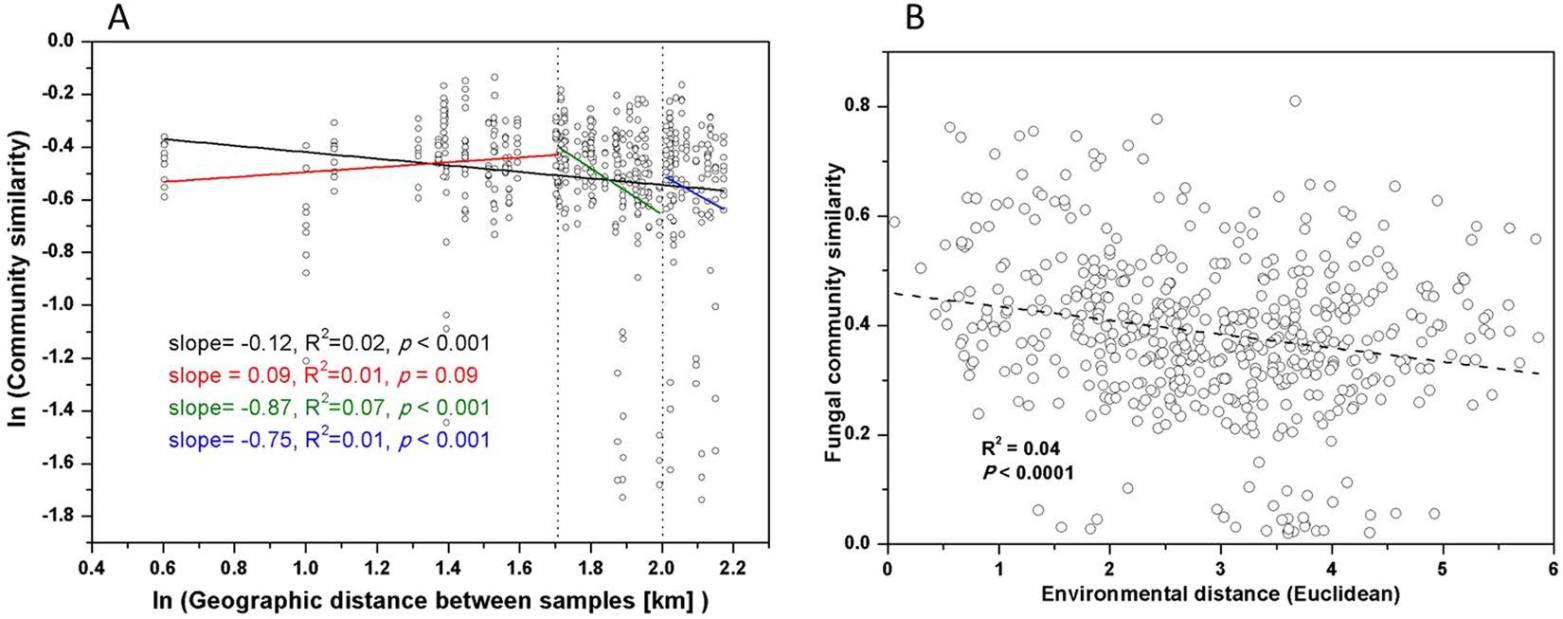
Distance-decay curves for the fungal communities. (**A**) Distance-decay for fungal community along geographical distance. The black line denotes the linear regression across all spatial scales. The colorful lines indicate separate regressions within each of the three spatial scales: 0–50 km, 50–100 km, 100–150 km. The slopes of all lines (except the solid red blue line) are significantly less than zero. The slopes of the green and blue lines are significantly different from the slope of the all scale (black solid) line. (**B**) Distance-decay for fungal community along environmental distance.

A plot of community similarity against geographic distance and environmental distance for each pairwise set of fungal samples revealed a significant distance decay relationship (Fig. 4). Furthermore, the slope of geographic distance decay curve varied significantly among the three spatial scales. The distance-decay slope was steeper (slope = −0.87, *P* < 0.001) in an intermediate range between 50 to 100 km than a range between 100 to 150 km (slope = −0.75, *P* < 0.001) and the overall slope (slope = −0.12, *P* < 0.001) (Fig. 4). In contrast, the distance-decay curve did not differ from zero at the smaller scale (*P* = 0.09). Multivariate Mantel correlograms showed that pairwise comparisons of fungal community composition were autocorrelated at a distance of less than approximately 60 km (Figure S1). Mantel tests indicated that the community of fungi was significantly correlated with either environmental or geographical distance (Table 2), where environmental distance accounted for a higher proportion of variation in community structure. However, partial Mantel tests indicated that the fungal community was significantly correlated with environmental distance, but not with geographic distance (Table 2).

**Table 2:** Results of Mantel and partial Mantel tests for the correlation between fungal community similarity and environmental distance (Euclidean), and between fungal community similarity and geographical distance. Statistical significance for each partial Mantel correlation value is given in parentheses.

	Mantel test	Partial Mantel test
Environmental distance	**−0**.**21(0**.**021)**	**−0**.**15(0**.**046)**
Geographical distance	**−0**.**17(0**.**014)**	−0.09(0.10)

**Figure 5:**
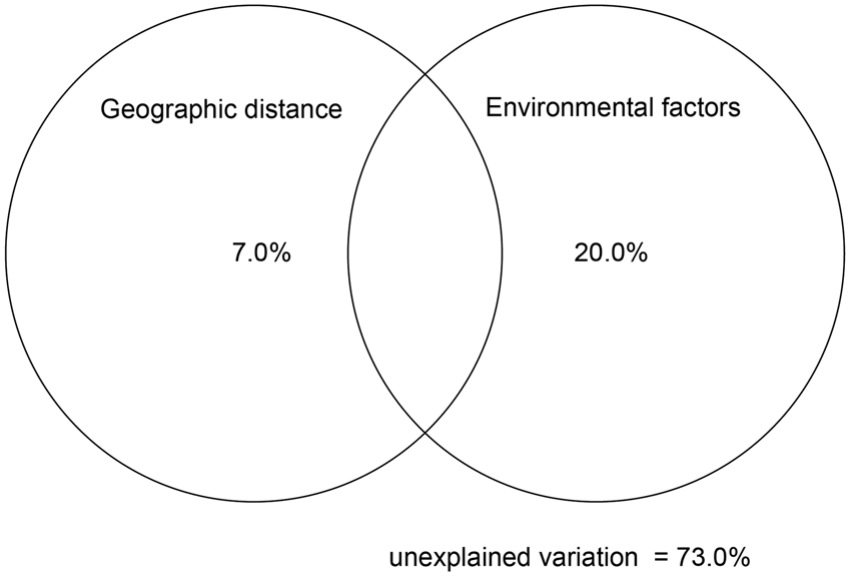
Variation partition analysis to determine an effect of geographic distance and environmental variables on the fungal communities.

Variance partitioning analysis (VPA) was performed to quantify the relative contributions of geographic distance and sediment environmental parameters on the taxonomic structure of the fungal communities. Combination of a subset of environmental parameters (DOC, MAT, TN and C/N ratio) and geographic distance explained 27% of the observed variation, leaving 73% of the variation unexplained. The sediment properties explained 20.0% (P = 0.001), and geographic distance alone explained 7.0% of the variations (P = 0.03), and no interaction effect was detected (Fig. 5). Although the sediment properties together explained more of the variation, geographic distance by itself explained 12.2% of the observed variation, more than any of the other four of the individual sediment variables (Fig. 5). Thus both environmental factors and geographic distance are important factors in shaping bacterial community structures.

## Discussion

Sediment fungal diversity is intuitively thought to be low in aquatic habitats due to uniform vegetation and the physiological constraints of submersion in water^34^. In our study, unexpectedly high fungal diversity was detected in the sediments of HRYR which is consistence with recent study on fungal communities in sediments^35^. We found the fungal classes Sordariomycetes, Agaricomycetes, Dothideomycetes, Leotiomycetes and Pezizomycetes in large proportions, consistent with previous studies^35–38^. Most of these dominant classes are terrestrial in natural, but also capable of being amphibious^9,20,25^. The majority of Sordariomycetes members are terrestrial and frequently detected near shores of tropical and subtropical freshwater ecosystems^39,40^. Most of these fungi break down lignin and cellulose from plant debris in the land–water interface^41^. The fungal order Polyporales, belonging to the class Agaricomycetes, was highly abundant; these are efficient terrestrial wood-decay fungi that play an important role in the carbon cycle^42^. Polyporales can be introduced into lakes through high fungal propagule loads via inflowing streams, rainwater, and wind^35,43^. The expansion of desaturase-encoding genes in the Polyporales may help them easily adapt to the substrate and colonize succession series^42,44^. Dothideomycetes, and particularly the order Pleosporales are found in many low-temperature lakes^45^, due to their resistance or adaptation to low temperature^37^, which is essential for survival in the harsh environment of the HRYR. These findings have implications for microbial biogeography, suggesting that in such habitat, environmental selection may be a more important determinant of community composition than dispersal constraints^2^.

At the spatial extent of our study, although both environmental characteristics and geographic proximity was significantly related to fungal community similarity, the partial correlation coefficient indicated that environmental filtering had a greater influence on fungal community. In our study, fungal community similarity was controlled by environmental factors such as TN and MAT. Nitrogen availability affects the fungal growth^46–48^ and may limit the fungal diversity in the soil^49,50^. The QTPs’ low temperatures lead to lake fungal community structure in the HRYR similar to what is observed in polar regions by Cox *et al*.^2^ and Newsham *et al*.^51^. Marginal result of increasing fungal diversity with warming temperature may result from enhanced metabolic activity and enable a switch from survival to growth and dispersal strategies^51^. Tian *et al*.^52^ identified temperature as one of major factor regulating fungal community structure in Qinghai-Tibetan Plateau. Hence, low temperature as an environmental constraint may determine fungal community composition in these extreme habitats. Interestingly, sediments pH significantly affected the fungal alpha diversity but not the fungal community similarity, which is consistence with report of Wang *et al*.^53^. With an increasing pH, some endurable taxa started to grow, resulting in an increase of the community richness. Although, we measured the impact of various environmental factors known or assumed to be an important in shaping fungal communities, but we were unable to explain all the variation. The unexplained variation may result from other unmeasured factors that could have an effect on fungal assemblages.

Distance–decay data can be used to understand the factors driving community turnover patterns such as dispersal limitation and environmental heterogeneity^54^. In this study, the slope of geographic distance-decay relationship (slope = −0.12, *P* < 0.001) was similar or steeper compared to the other reported studies^5,55,56^, indicating that dispersal limitation was also important in shaping fungal community. However, there was no significant distance-decay relationship observed at less than 50 km scale, indicates that dispersal limitation was less important in shaping fungal community. One plausible explanation is that dispersal should be more efficient at this spatial scales since long-distance dispersal of fungi mainly occur by wind^57^, resulting in weaker spatial structure of the communities and local control by environmental filtering^29^. Furthermore, in the QTP, there is more area dominated by saline lakes than freshwater lakes. Since the saline lakes are relatively isolated from large river basins and thus are less hydrologically connected^58^, it would be expected that dispersal limitation has a stronger control on fungal community in the saline lakes than in freshwater ones.

In conclusion, a large proportion of fungal sequences obtained in high frequency matched with terrestrial taxa, indicating that they are transported into the aquatic system and subsequently adapt to the local conditions. Mantel tests and variance partitioning analysis suggested that fungal richness and community composition are strongly influenced by environmental factors such as temperature and TN. Nonetheless, a significant spatial distance decay relationship and widespread locally abundant fungi indicated that dispersal limitation also plays a role in shaping fungal community. Different patterns of diversification might be observed depending on the ecology of the studied taxa. It is therefore important to investigate organisms that are typical for each biome occurring in the region of the QTP.

## Methods and Materials

### Sampling site

We selected 10 freshwater lakes with areas ranging from 3 to 611 km^2^ in the HRYR, including Lake Erling, the largest lake in this region (Table 1). Sampling map was generated by ArcGIS software under license. The minimal and maximal distance between any two lakes were 4 and 148 km, respectively (Fig. 1 and Table 1). Most of the lakes were not readily interconnected to each other via water routes. The dominant vegetation type in the catchments of the 10 lakes was alpine grassland.

### Sampling collection

We collected surface sediments up to 15 cm deep from inshore sites with water depths of approximately 1 m from each of the 10 freshwater lakes (Fig. 1). Three samples, spaced between 3 and 5 m, were collected from each lake. Samples were kept at 4 °C for sediment geochemical characterization and stored at −80 °C for genomic DNA extraction. Global positioning system coordinates were recorded at each sampling point (Table 1), and imported into the NOAA website (http://www.nhc.noaa.gov/gccalc.shtml) to calculate the pairwise geographic distance. The air temperature was measured in the field. Local mean annual temperature (MAT) was obtained from local weather stations. The pH was determined with a soil to water ratio of 1:5 wt/vol. Dissolved organic carbon (DOC) and total nitrogen (TN) were measured with a vario TOC cube analyzer (ELEMENTAR Analysensysteme, Germany). Before analyzing sediment DOC, samples were first acidified with 1 N HCl overnight to remove carbonates and subsequently washed to neutralize pH, dried in an oven and then ground in a mortar.

### DNA extraction, PCR and pyrosequencing

Total genomic DNA for 454 Life Sciences sequencing was extracted from 0.5 g of frozen soil with the FastDNA™ SPIN Kit for Soil DNA (MP Biomedicals, USA) according to manufacturer’s instructions. The obtained DNA was quantified by a NanoDrop device and stored at −20 °C. An aliquot of 50 ng purified DNA from each sample was used as a template for amplification. A set of primers was designed by adding a Roche 454 ‘A’ pyrosequencing adapter and adding a unique 8-bp barcode sequence to forward primer ITS1F (5′-CTTGGTCATTTAGAGGAAGTAA-3′) and adding reverse primer ITS2 (5′-GCTGCGTTCTTCATCGATGC-3′) to generate polymerase chain reaction (PCR) ITS1 region fragments of *c*. 400 bp. Each sample was amplified in triplicate with 50 µl reactions under the following conditions: 94 °C for 4 min, 30 cycles of 30 s at 94 °C (denaturation), 50 °C for 1 min (annealing) and 72 °C for 90 s (extension), followed by 10 min at 72 °C. PCR products were pooled together and purified by an Agarose Gel DNA purification kit (TaKaRa, China) and quantified with the NanoDrop device. Samples were run on a ROCHE 454 FLX + platform (Roche, Basel, Switzerland) at the National Human Genome Center of China in Shanghai, China, according to the manufacturer’s manual. These raw data are deposited into the NCBI Sequence Read Archive with accession number SRP108149.

### Sequence analysis

The raw sequence data were demultiplexed using the QIIME toolkit^59^. Sequence reads with a quality score less than 20 were filtered out (split_libraries_fastq.py; QIIME), and the remaining reads were assigned to samples according to the corresponding barcode. The extracted sequences were binned into operational taxonomic units (OTUs) at 97% match using USEARCH^60^, denoting a species-level similarity using closed-reference OTU picking using the UNITE ITS database^61^ as a reference (pick_otus.py; QIIME). One representative of each OTU was selected for downstream analyses, and a taxonomy was assigned to each (pick_rep_set.py, assign_taxonomy.py; QIIME).

### Statistical analyses

Singletons were removed prior to statistical analyses. Diversity indices Chao1, ACE and Shannon (H′) were estimated based on the OTU table rarefied at 3229 counts per sample. We used linear and quadratic regression to test whether fungal diversity was related to lake area and environmental variables (TN, pH, DOC and MAT).

Distance decay of similarity has been widely used to infer the relative importance of dispersal limitation and environmental filtering in structuring microbial community^62^. To determine the distance decay relationship, pairwise fungal community similarities between samples were calculated using the Sørensen index, which is widely used for calculating distance decay in both micro- and macroecology. The spatial distance decay relationship was then assessed as the slope of logarithmic space to enhance the linear fitting, according to the description of Nekola & White (1999)^63^. All logarithmic transformations were performed using the natural logarithm. Mantel tests were performed to examine community turnover along environmental and geographical gradients. Euclidean distances were calculated for all site pairs separately based on the environmental variables and lake coordinates. Environmental data was comprised of total N, DOC, pH, water content and temperature. A significant correlation was observed between environmental and geographic distance in our study (Mantel r = 0.41, *P* = 0.001) (Fig. S2). Therefore, we performed partial Mantel tests (with 1000 permutations) to disentangle the effects of environmental distance on fungal community similarity while holding geographical distance constant, and vice versa^30^.

To fully characterize the exact scale of spatial clustering, a multivariate Mantel correlogram analysis was performed using the ‘*mantel*.*correlog*’ command in the R software. A Mantel correlogram draws a graph in which Mantel correlation values *r* are plotted as a function of the geographic distance classes among the sampling sites.

BioEnv and distance-based redundancy analysis (db-RDA) were also used to identify the abiotic factors influencing fungal community composition, and subsequently used to construct the sediment property matrix for variation partitioning analysis. Variance was partitioned to examine the relative effect of geographic distance and environmental variables (best environmental subset) on microbial community composition. All of the above analyses were performed in the *vegan* package in R software.

## Electronic supplementary material

supplementary information

## References

[1] Fukami T (2010). Assembly history dictates ecosystem functioning: evidence from wood decomposer communities. Ecol. Lett..

[2] Cox F, Newsham KK, Bol R, Dungait JAJ, Robinson CH (2016). Not poles apart: Antarctic soil fungal communities show similarities to those of the distant Arctic. Ecol. Lett..

[3] Favre A (2015). The role of the uplift of the Qinghai-Tibetan Plateau for the evolution of Tibetan biotas. Biol. Rev..

[4] Ferrari BC (2016). Geological connectivity drives microbial community structure and connectivity in polar, terrestrial ecosystems. Environ. Microbiol..

[5] Bahram M, Peay KG, Tedersoo L (2015). Local-scale biogeography and spatiotemporal variability in communities of mycorrhizal fungi. New Phytol..

[6] Kivlin SN, Winston GC, Goulden ML, Treseder KK (2014). Environmental filtering affects soil fungal community composition more than dispersal limitation at regional scales. Fungal Ecol..

[7] Green JL (2004). Spatial scaling of microbial eukaryote diversity. Nature.

[8] Tedersoo L (2014). Global diversity and geography of soil fungi. Science.

[9] Crump BC, Amaral-Zettler LA, Kling GW (2012). Microbial diversity in arctic freshwaters is structured by inoculation of microbes from soils. ISME J..

[10] Treseder KK (2014). Evolutionary histories of soil fungi are reflected in their large-scale biogeography. Ecol. Lett..

[11] Peay KG, Garbelotto M, Bruns TD (2010). Evidence of dispersal limitation in soil microorganisms: Isolation reduces species richness on mycorrhizal tree islands. Ecology.

[12] Davison J (2015). Global assessment of arbuscular mycorrhizal fungus diversity reveals very low endemism. Science.

[13] Feinstein LM, Blackwood CB (2013). The spatial scaling of saprotrophic fungal beta diversity in decomposing leaves. Mol. Ecol..

[14] Kivlin SN, Hawkes CV, Treseder KK (2011). Global diversity and distribution of arbuscular mycorrhizal fungi. Soil Biol. Biochem..

[15] White WR, Crisman TL (2016). Headwater Streams of Florida: Types, Distribution and a Framework for Conservation. River Res. Appl..

[16] Meyer JL (2007). The contribution of headwater streams to biodiversity in river networks. J. Am. Water Resour. Assoc..

[17] Clarke A, Mac Nally R, Bond N, Lake PS (2008). Macroinvertebrate diversity in headwater streams: a review. Freshwater Biol..

[18] Nino-Garcia JP, Ruiz-Gonzalez C, del Giorgio PA (2016). Interactions between hydrology and water chemistry shape bacterioplankton biogeography across boreal freshwater networks. ISME J..

[19] Ruiz-Gonzalez C, Nino-Garcia JP, del Giorgio PA (2015). Terrestrial origin of bacterial communities in complex boreal freshwater networks. Ecol. Lett..

[20] Besemer K (2013). Headwaters are critical reservoirs of microbial diversity for fluvial networks. Proc. R. Soc. B..

[21] Finn DS, Bonada N, Murria C, Hughes JM (2011). Small but mighty: headwaters are vital to stream network biodiversity at two levels of organization. J. N. Amer. Benthol. Soc..

[22] Carrara F, Altermatt F, Rodriguez-Iturbe I, Rinaldo A (2012). Dendritic connectivity controls biodiversity patterns in experimental metacommunities. Proc. Natl. Acad. Sci. USA.

[23] Brown BL (2011). Metacommunity theory as a multispecies, multiscale framework for studying the influence of river network structure on riverine communities and ecosystems. J. N. Amer. Benthol. Soc..

[24] Hauptmann AL (2016). Upstream Freshwater and Terrestrial Sources Are Differentially Reflected in the Bacterial Community Structure along a Small Arctic River and Its Estuary. Front. Microbiol..

[25] Nelson CE, Sadro S, Melack JM (2009). Contrasting the influences of stream inputs and landscape position on bacterioplankton community structure and dissolved organic matter composition in high- elevation lake chains. Limnol. Oceanogr..

[26] Hu GY (2017). Holocene aeolian activity in the Headwater Region of the Yellow River, Northeast Tibet Plateau, China: A first approach by using OSL-dating. Catena.

[27] Duan S, Fan S, Cao G, Liu X, Sun Y (2015). The changing features and cause analysis of the lakes in the source regions of the Yellow River from 1976 to 2014. J. Glaciol. Geocryol..

[28] Rosati, M. *et al*. Are aquatic assemblages from small water bodies more stochastic in dryer climates? An analysis of ostracod spring metacommunities. *Hydrobiologia*, 1–14 (2016).

[29] Soininen J, Korhonen JJ, Karhu J, Vetterli A (2011). Disentangling the spatial patterns in community composition of prokaryotic and eukaryotic lake plankton. Limnol. Oceanogr..

[30] Teittinen A, Soininen J (2015). Testing the theory of island biogeography for microorganisms-patterns for spring diatoms. Aquat. Microb. Ecol..

[31] Heino J (2015). Metacommunity organisation, spatial extent and dispersal in aquatic systems: patterns, processes and prospects. Freshwater Biol..

[32] Zhang JX (2015). Distribution of sediment bacterial and archaeal communities in plateau freshwater lakes. Appl. Microbiol. Biotechnol..

[33] Xiong J (2012). Geographic distance and pH drive bacterial distribution in alkaline lake sediments across Tibetan Plateau. Environ. Microbiol..

[34] Shearer CA (2007). Fungal biodiversity in aquatic habitats. Biodivers. Conserv..

[35] Li W (2016). Fungal communities in sediments of subtropical Chinese seas as estimated by DNA metabarcoding. Sci. Rep..

[36] Zhang LK, Kang MY, Huang YC, Yang LX (2016). Fungal communities from the calcareous deep-sea sediments in the Southwest India Ridge revealed by Illumina sequencing technology. World J. Microb. Biot..

[37] Nagano Y, Nagahama T (2012). Fungal diversity in deep-sea extreme environments. Fungal Ecol..

[38] Arfi Y, Marchand C, Wartel M, Record E (2012). Fungal diversity in anoxic-sulfidic sediments in a mangrove soil. Fungal Ecol..

[39] Matsuoka S, Kawaguchi E, Osono T (2016). Temporal distance decay of similarity of ectomycorrhizal fungal community composition in a subtropical evergreen forest in Japan. FEMS Microbiol. Ecol..

[40] Tsui CKM, Hyde KD, Hodgkiss IJ (2000). Biodiversity of fungi on submerged wood in Hong Kong streams. Aquat. Microb. Ecol..

[41] Zhang N (2006). An overview of the systematics of the Sordariomycetes based on a four-gene phylogeny. Mycologia.

[42] Binder M (2013). Phylogenetic and phylogenomic overview of the Polyporales. Mycologia.

[43] Wurzbacher CM, Barlocher F, Grossart HP (2010). Fungi in lake ecosystems. Aquat. Microb. Ecol..

[44] Fernandez-Fueyo E (2012). Comparative genomics of Ceriporiopsis subvermispora and Phanerochaete chrysosporium provide insight into selective ligninolysis. Proc. Natl. Acad. Sci. USA.

[45] Gonçalves VN, Vaz ABM, Rosa CA, Rosa LH (2012). Diversity and distribution of fungal communities in lakes of Antarctica. FEMS Microbiol. Ecol..

[46] Siciliano SD (2014). Soil fertility is associated with fungal and bacterial richness, whereas pH is associated with community composition in polar soil microbial communities. Soil Biol. Biochem..

[47] Meng H (2013). Responses of bacterial and fungal communities to an elevation gradient in a subtropical montane forest of China. Appl. Microbiol. Biotechnol..

[48] Beauregard MS, Hamel C, Atul N, St-Arnaud M (2010). Long-Term Phosphorus Fertilization Impacts Soil Fungal and Bacterial Diversity but not AM Fungal Community in Alfalfa. Microb. Ecol..

[49] Cox F, Barsoum N, Lilleskov EA, Bidartondo MI (2010). Nitrogen availability is a primary determinant of conifer mycorrhizas across complex environmental gradients. Ecol. Lett..

[50] Allison SD, Hanson CA, Treseder KK (2007). Nitrogen fertilization reduces diversity and alters community structure of active fungi in boreal ecosystems. Soil Biol. Biochem..

[51] Newsham KK (2016). Relationship between soil fungal diversity and temperature in the maritime Antarctic. Nat. Clim. Change.

[52] Tian JQ (2017). Patterns and drivers of fungal diversity along an altitudinal gradient on Mount Gongga, China. J. Soils Sediments.

[53] Wang J-T (2015). Soil pH determines the alpha diversity but not beta diversity of soil fungal community along altitude in a typical Tibetan forest ecosystem. J. Soils Sediments.

[54] Ferrier S, Manion G, Elith J, Richardson K (2007). Using generalized dissimilarity modelling to analyse and predict patterns of beta diversity in regional biodiversity assessment. Divers. Distrib..

[55] Bahram M (2013). The distance decay of similarity in communities of ectomycorrhizal fungi in different ecosystems and scales. J. Ecol..

[56] Zinger L, Boetius A, Ramette A (2014). Bacterial taxa-area and distance-decay relationships in marine environments. Mol. Ecol..

[57] Peay KG, Bruns TD (2014). Spore dispersal of basidiomycete fungi at the landscape scale is driven by stochastic and deterministic processes and generates variability in plant-fungal interactions. New Phytol..

[58] Jiang J, Huang Q (2004). Distribution and variation of lakes in Tibetan Plateau and their comparison with lakes in other part of China. Water Resour. Prot..

[59] Caporaso JG (2010). QIIME allows analysis of high-throughput community sequencing data. Nature Methods.

[60] Edgar RC, Haas BJ, Clemente JC, Quince C, Knight R (2011). UCHIME improves sensitivity and speed of chimera detection. Bioinformatics.

[61] Koljalg U (2005). UNITE: a database providing web-based methods for the molecular identification of ectomycorrhizal fungi. New Phytol..

[62] Gilbert B, Lechowicz MJ (2004). Neutrality, niches, and dispersal in a temperate forest understory. Proc. Natl. Acad. Sci. USA.

[63] Nekola JC, White PS (1999). The distance decay of similarity in biogeography and ecology. J. Biogeogr..

