# Supplementary material for: Environmental factors driving fungal distribution in freshwater lake sediments across the Headwater Region of the Yellow River, China
Source: Sci Rep. 2018 Feb 28;8:3768. doi: 10.1038/s41598-018-21995-6 (PMC5830880; doi:10.1038/s41598-018-21995-6)

**Environmental factors driving fungal distribution in freshwater lake sediments across the Headwater Region of the Yellow River, China**

Jianqing Tian^1#^, Dan Zhu^2,3#^, Jinzhi Wang^4^, Bing Wu^1^, Muzammil Hussain^1^，Xingzhong Liu^1*^

^1^State Key Laboratory of Mycology, Institute of Microbiology, Chinese Academy of Sciences, Beijing, 100101, China

^2^CAS Key Laboratory of Mountain Ecological Restoration and Bioresource Utilization & Ecological Restoration and Biodiversity Conservation Key Laboratory of Sichuan Province, Chengdu Institute of Biology, Chinese Academy of Sciences, Chengdu, 610041, China

^3^Zoige Peatland and Global Change Research Station, Chinese Academy of Sciences, Hongyuan, 624400, China

^4^Beijing Key Laboratory of Wetland Services and Restoration, Institute of Wetland Research, Chinese Academy of Forestry, Beijing 100091, China

# These authors contributed equally to this work.

*Corresponding author: Xingzhong Liu ([liuxz@im.ac.cn](mailto:liuxz@im.ac.cn))

**Figure S1** A Mantel correlogram shows the extent of spatial autocorrelation of the sediments fungal community composition as a function of geographic isolation (m). A positive Mantel correlation indicates greater community dissimilarity with increasing geographic distance separating the communities. Solid black square represent scales with statistically significant spatial autocorrelation


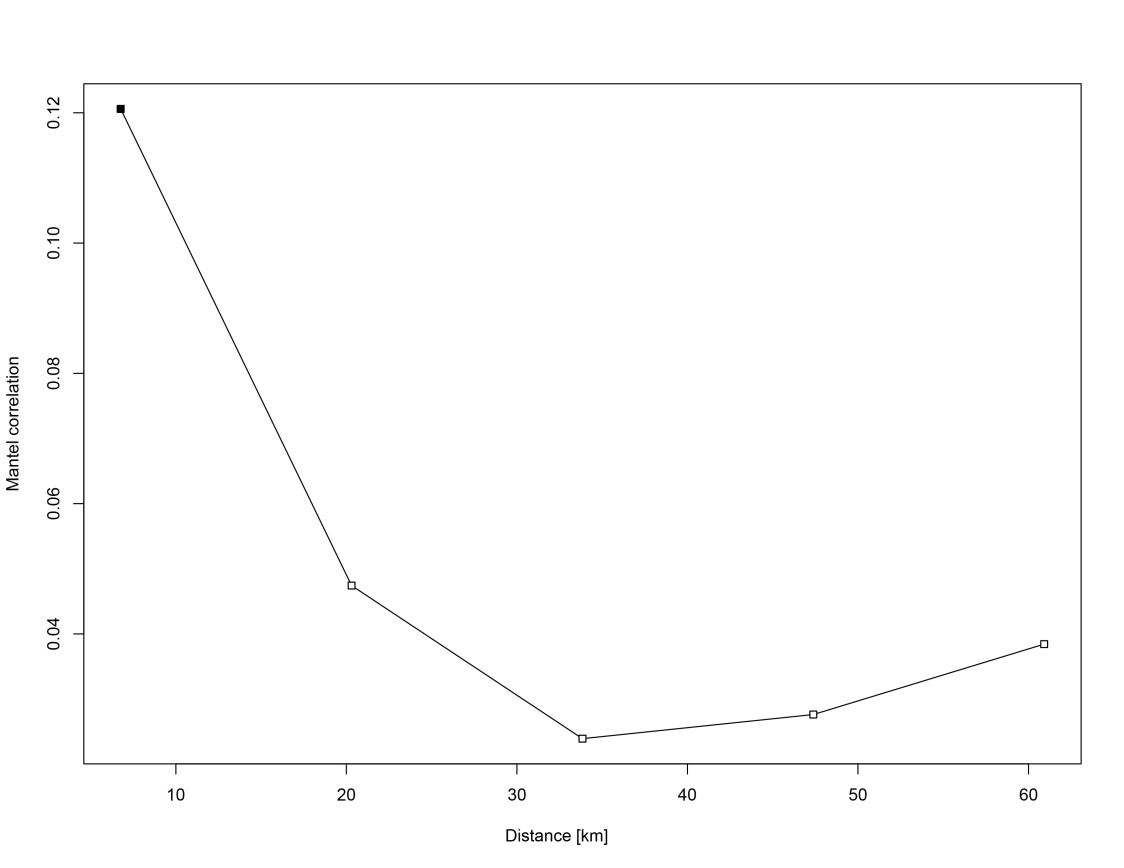


**Figure S2** Relationship between geographic and environmental distance between all site pairs (Mantel correlation = 0.41, P = 0.001).


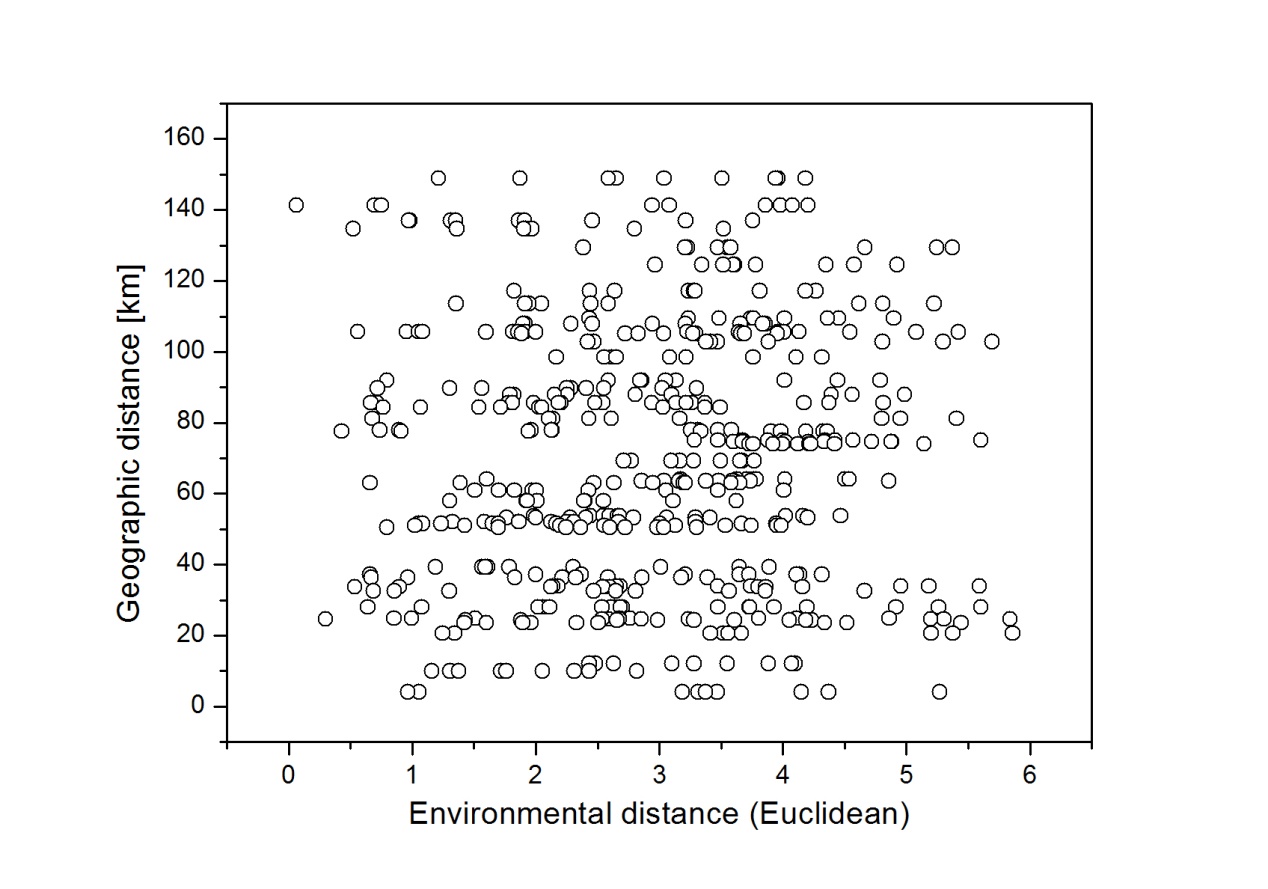

Supplement: Supplementary file 1 — supplementary information [file 41598_2018_21995_MOESM1_ESM.doc]
